# Factors influencing Ontario dairy veterinarians’ management and care of down dairy cows

**DOI:** 10.3389/fvets.2025.1519284

**Published:** 2025-03-04

**Authors:** John E. Brindle, David L. Renaud, Derek B. Haley, Todd F. Duffield, Charlotte B. Winder

**Affiliations:** ^1^Department of Population Medicine, University of Guelph, Guelph, ON, Canada; ^2^Campbell Centre for the Study of Animal Welfare, University of Guelph, Guelph, ON, Canada

**Keywords:** non-ambulatory, management practices, recumbent, veterinary, recommendations

## Abstract

This cross-sectional study assessed what management practices veterinarians recommended for down dairy cows in Ontario, Canada, and identified factors influencing producers’ adoption of protocols. An online survey about veterinary involvement in down cow management was available between February and May 2021, distributed by email through the Ontario Association of Bovine Practitioners (OABP). A total of 48 Ontario bovine veterinarians responded (26.8% response rate). Gender distribution was even between those identifying as male or female (50%), and the majority of respondents were between 30 to 39 years old. Veterinarians most commonly suggested housing down dairy cows in individual pens (40.7%), followed by pasture (29.6%), special pens for three or fewer animals (26%), and special pens for four or more animals (3.7%). Regarding spacing allowance for a down dairy cow, many veterinarians suggested 11.1 (120) to 23.2 (120–250 square feet) square meters (53.3%) per cow. Recommendations for moving down dairy cows included using a sled (62.5%), stone boat (56.3%), front-end loader bucket (45.8%), wheeled cart (20.8%), and hip-lifter (2.1%). For lifting down dairy cows, recommendations included using multiband slings (56.2%), hip lifters (43.8%), floatation tanks (25.0%), single belly slings (14.6%), ropes (4.2%), and hip lifters with additional straps (2.1%). A multivariable linear regression model identified key factors associated with the recommended time to assist a down cow to stand. Specifically, veterinarians who spent over 90% of their working hours annually with dairy cattle recommended assisting cows 14.1 h earlier than those who spent less than 85% of their time working with dairy cattle. Additionally, larger clinics advised waiting 12.4 h longer compared to smaller clinics, and veterinarians who recommended waiting 12–24 h before calling a veterinarian suggested assisting cows 13.8 h later than those recommending a wait of less than 7 h. Implementing a more consistent, evidence-based approach by veterinarians could enhance the care of down dairy cows and support the broader objective of improving management protocols.

## Introduction

Non-ambulatory (“down”) dairy cattle are a challenge for the dairy industry, a problem that may be compounded by the variety of definitions, presentations, and diverse range of contributing factors (1). North American quality assurance programs in the dairy industry, such as proAction® and Farmers Assuring Responsible Management (FARM), have specific requirements addressing down dairy cow management (2, 3); however, despite these requirements, improvements are still needed to ensure that the minimum standards are met (4). There is evidence that cows are often treated at or below these requirements with poor quality nursing care, which can result in additional injury and a worse prognosis for recovery (5, 6). According to Poulton et al., high-quality nursing care for down cows can be defined as attentive and proactive management that minimizes secondary injuries, promotes comfort, and supports recovery through frequent repositioning, clean bedding, and prompt treatment of complications. In order to improve animal welfare and outcomes for down dairy cows, developing appropriate care plans are needed. In many cases, these plans will be developed in consultation with the herd veterinarian, as it ensures adherence to regulations, such as those outlined in proAction and FARM (2, 3).

Veterinarians are important in providing advice and guidance to producers for improving dairy cow welfare and health (7–9). Producers frequently seek their veterinarian’s advice on welfare-related issues, particularly to increase their awareness of these concerns (10). Additionally, veterinarians can play a role in promoting behavior change among farmers (11) by fostering a shared understanding with producers, leading to increased trust and receptiveness to new practices (7, 11). By comprehending producers’ decision-making processes, veterinarians can tailor best management practices to individual farm needs, ultimately promoting positive change and enhancing dairy cow health and welfare (8, 11).

While some studies have examined management practices implemented for down dairy cows on dairy farms (5, 12, 13), to our knowledge none have surveyed veterinarians’ recommendations for these. A better understanding of veterinarians’ recommendations and influence in the decision-making process of producers regarding the management of down dairy cows is needed (14). Hence, the objective of this cross-sectional study was to assess what management practices veterinarians recommended for down dairy cows in Ontario, Canada and assess the factors associated with the recommended time to assist a cow to stand and calling the veterinarian.

## Materials and methods

This study is reported following the Strengthening the Reporting of Observational Studies in Epidemiology (Veterinary Extension) (STROBE-VET) guidelines (15). Prior to beginning the study, human subject ethical approval was obtained from the University of Guelph (Ontario, Canada) (REB#20–09-001).

### Questionnaire development

A web-based survey was available from February to May 2021 to examine the prevailing expectations, recommendations, and viewpoints of Ontario bovine veterinarians for five management areas: cull cows, down cows, male and female dairy calves, antimicrobial use, and disease control and surveillance. The results from the survey section about cull cows have been published (16). The survey also collected demographic information about the veterinarians and the clinics where they worked. The survey was developed in collaboration with several researchers from the Ontario Veterinary College specializing in dairy cattle health and welfare. A pretest of the survey was conducted with three recently retired bovine veterinarians, with no changes made to the survey after their review. Recruitment of respondents was conducted through email newsletters distributed by the Ontario Association of Bovine Practitioners (OABP). The survey was only distributed once with two follow-up emails. An incentive of a $10 gift card was offered to the first 10 respondents.

Eligibility criteria for participation consisted of respondents being actively practicing veterinarians in Ontario and having worked with dairy cattle in the past 12 months. The survey, which encompassed 75 questions (38 pertained to down cow management), was conducted as an online questionnaire (Qualtrics; Utah, United States). All supplemental material including the questionnaire can be found at the following link.^1^ The survey took approximately 30 min to complete. Various question types, such as multiple choice, Likert scale, sliding scales, ranking, and open-ended comment boxes, were incorporated. The survey design limited the number of questions presented per page and randomized multiple-choice questions within each section to help mitigate any possible bias due to order effects.

### Statistical analysis

The survey data were exported from Qualtrics (Qualtrics, Provo, UT) and imported into a Jupyter notebook (version 7) using the Python programming language (version 3.11) and the Pandas library (version 1.5.2) for data processing. Data were checked for errors and completeness. Respondents could skip questions or end the survey anytime, resulting in some unanswered questions. Stata (version 17, StataCorp LLC, College Station, TX) and Python (version 3.11) were used to calculate descriptive statistics for all explanatory variables. Categorical variables with low response rates (< 5 observations) were combined into larger categories for robust statistical analysis. Questions that contained an ‘other’ text box option were screened, and the responses were sorted into the appropriate category.

A multivariable linear regression model was built to assess the factors associated with the recommended time from identifying a cow as down to assisting her to standing, whereas several univariable linear regression models were built to explore the time from identifying the cow as down until calling the veterinarian. Univariable models were presented when there were no significant variables retained in the multivariable model. The linearity of the relationship between continuous predictors and the outcome variables were assessed using locally weighted scatterplot smoothing (lowess) generated with Stata 17. In cases where a non-linear relationship was identified, variables were categorized into quartiles. To avoid including collinear variables, a pairwise correlation matrix was examined, and variables with a correlation coefficient greater than 0.8 were considered collinear. In the event of collinearity, the variable with more missing responses was excluded.

Explanatory variables were screened using univariable regression for each outcome, and variables with *p* < 0.20 were considered for inclusion in the multivariable model. The model building followed a manual backward stepwise procedure, where only variables with *p* < 0.05, confounders, or those with a significant interaction term were included in the final model. Confounding was assessed by determining if a change of at least 25% occurred in a variable’s coefficient upon its removal, in which case the potential confounder was retained in the model. The standardized residuals of the linear models were assessed graphically and using a Shapiro–Wilk test where normality was assumed if the *α* was >0.05. Outliers were identified using plots of standardized residuals, leverage, delta-beta, Cook’s distance, and difference in fit (DFITS). For all tests, a *tendency* was defined as a notable deviation from the expected patterns or a substantial impact of individual observations, warranting further investigation and consideration in interpreting the results.

## Results

### Demographic and clinic characteristics

Demographic characteristics and clinic information of the surveyed veterinarians in Ontario are shown in Table 1 (categorical) and Table 2 (numerical). Of the total 179 currently practicing bovine veterinarians registered with the Ontario Association of Bovine Practitioners in 2021, 48 responded to the survey, resulting in a response rate of 26.8%. Gender distribution among respondents who identified their pronouns revealed an even split between he/him (*n* = 15) and she/her (*n* = 15), with 18 respondents choosing to not answer. The majority of respondents were between 30 and 39 years, graduated from the Ontario Veterinary College, and were located in Central Ontario.

**Table 1:** Number and proportion of different demographic and veterinarian characteristics reported by survey respondents in Ontario (*n* = 48).

Variable	n*	Proportion (%)
Age	46	
20 to 29 years		10/46 (21.7%)
30 to 39 years		19/46 (41.3%)
40 to 49 years		7/46 (15.2%)
50 to 59 years		7/46 (15.2%)
60 to 69 years		3/46 (6.5%)
Licensed OMAFRA sales barn inspector	45	
No		31/45 (68.9%)
Yes		14/45 (31.1%)
Practicing Ontario veterinarian	48	
No		3/48 (6.2%)
Yes		45/48 (93.8%)
Pronoun	30	
He/him		15/30 (50.0%)
She/her		15/30 (50.0%)
Clinic region	40	
Central Ontario		23/40 (57.5%)
Eastern Ontario		4/40 (10.0%)
Greater Toronto		7/40 (17.5%)
Southwestern Ontario		6/40 (15.0%)
Veterinary school	46	
Atlantic Veterinary College		3/46 (6.5%)
Ontario Veterinary College		40/46 (87.0%)
University College Dublin		3/46 (6.5%)

*n is the number of respondents answering the question. The survey was distributed to bovine veterinarians in Ontario and evaluated 5 management areas (cull cows, down cows, male and female dairy calves, antimicrobial use, and disease control and surveillance).

**Table 2:** Number, median, and range of different veterinarian and clinic characteristics reported by survey respondents in Ontario (*n* = 48).

Variable	n*	Median	Range
Graduation year	46	2011	1978–2020
Time spent working with species per year (h):	46		
Beef		8 h	0–75 h
Companion		0 h	0-55 h
Dairy		80 h	14-100 h
Equine		5 h	0–75 h
Small ruminant		3 h	0-20 h
Other (swine, poultry, and camelids)		0 h	0-10 h
Total number of dairy veterinarians at their clinic	41	4	1–13
Total number of dairy farms serviced by their clinic	41	60	5–400
Total number of regular health visits (monthly) per respondent	41	15	0–60
Any service (e.g., herd health) visits (monthly) per respondent	41	40	5–230
Average herd size of farms serviced by their clinic			
Dry cows	38	17.5	7–50
Lactating cows	39	80	50–160

*n is the number of respondents answering the question. The survey was distributed to bovine veterinarians in Ontario and evaluated 5 management areas (cull cows, down cows, male and female dairy calves, antimicrobial use, and disease control and surveillance).

The respondents worked in clinics employing a mean of 5 (Standard deviation ±3) veterinarians and the mean number of dairy farms serviced by clinics was 84 (± 86) farms. Respondents reported personally providing a mean of 57 (± 49) service visits/month including 18 (± 14) regular health visits per month. The average number of lactating cows on the farms serviced was 85 (± 25) and respondents reported working a median time of 80% with dairy farms ([range] 14–100%).

### Management of down cows

Respondents’ recommended management practices for down dairy cows are displayed in Table 3 (categorical) and Table 4 (numerical). Several questions in the survey allowed for multiple options (‘select all that apply’) meaning that the number reported may exceed the number of respondents.

**Table 3:** Number and proportion of different veterinarian recommendations reported for a down cow by survey respondents in Ontario (n = 48).

Variable	n*	Proportion (%)
Recommended reposition frequency (flipped from left to right)	30	
Once every 1–6 h		22/30 (73.3%)
Once every 7–12 h		8/30 (26.7%)
Recommended Frequency of delivery of feed and water	29	
2 times per day		2/29 (6.9%)
3 times per day		9/29 (31.0%)
4 times per day		3/29 (10.3%)
5 or more times per day		1/29 (3.4%)
Always available		14/29 (48.3%)
Recommended support therapy	48	
Anti-inflammatory drugs		30/48 (62.5%)
Fluid therapy		16/48 (33.3%)
Nutritional supplement		10/48 (20.8%)
Repositioning therapy		3/48 (6.2%)
Bloodwork		2/48 (4.2%)
Antibiotics		1/48 (2.1%)
Lifting therapy		1/48 (2.1%)
Physical exam		1/48 (2.1%)
Move methods considered appropriate	48	
Sled		30/48 (62.5%)
Stone boat		37/48 (56.2%)
Front-end loader bucket		22/48 (45.8%)
Wheeled cart		10/48 (20.8%)
Hip lifters		1/48 (2.1%)
Lift method recommendations	48	
Multiband slings		27/48 (56.2%)
Hip lifters		21/48 (43.8%)
Floatation tank		12/48 (25.0%)
Single band slings		7/48 (14.6%)
Ropes		2/48 (4.2%)
Hip lifters with straps		1/48 (2.1%)
Housing considered appropriate	48	
Individual pen		22/48 (45.8%)
Pasture		16/48 (33.3%)
Pen with 3 or less animals		14/48 (29.2%)
Pen with 4 or more animals		2/28 (4.2%)
Bedding considered appropriate	48	
Sand		30/48 (62.5%)
Straw		28/48 (58.3%)
Pasture		26/48 (54.2%)
Compost pack		10/48 (20.8%)
Rubber mats		1/48 (2.1%)
Recommended housing space for a down cow**	30	
100sqft		9/30 (30.0%)
120sqft		7/30 (23.3%)
150sqft		5/30 (16.7%)
200sqft		2/30 (6.7%)
250sqft		2/30 (6.7%)
300sqft		1/30 (3.3%)
400sqft		2/30 (6.7%)
Space to lunge		2/30 (6.7%)
Recommended laying position	48	
On her chest (supported)		24/48 (50.0%)
On her chest (unsupported)		15/48 (31.0%)
Recommended feed methods	48	
Within reach of the cow		28/48 (58.3%)
Loose on the floor		23/48 (47.9%)
Unsecured buckets or feeders		22/48 (45.8%)
Secured buckets or feeders		8/48 (16.7%)
Recommended water methods	48	
Within reach of the cow		26/48 (54.2%)
Unsecured buckets		23/48 (47.9%)
Secured buckets		17/48 (35.4%)
By hand		2/48 (4.2%)

*n is the number of respondents answering the question. **Meters squared: 9.29 m2, 11.15 m2,13.94 m2, 18.58 m2, 23.23 m2, 27.87 m2, 37.16 m2. The survey was distributed to bovine veterinarians in Ontario and evaluated 5 management areas (cull cows, down cows, male and female dairy calves, antimicrobial use, and disease control and surveillance). Question included ‘select all that apply’, thus the proportion may be greater than 100%.

**Table 4:** Number, median, and range of different management characteristics reported by survey respondents in Ontario (*n* = 48).

Variable	*n*	Median	Range
Proportion of veterinarian’s dairy clients who had their veterinarian:* (%)			
View down cow SOP	29	72	0–100
Provide input for developing down cow SOP	28	50	0–91
Provide input for updating down cow SOP	28	30	0–100
Time considered appropriate before euthanizing for (d):			
Nursing care**	30	3	2–10
Lifting***	28	3	0-7
Recommended time to lift a non-ambulatory cow from finder her down (m/h):			
Ropes (m)	1	35	35
Hip lifter (m)	19	5	1–35
Belly sling (m)	7	5	1–15
Multiband sling (m)	26	15.5	3–45
Floatation tank (h)	11	2	0–8
Recommended lifting frequency per day for non-ambulatory cows (d)			
Rope frequency	2	2.5	2–3
Hip lifter frequency	21	2	1–3
Single band sling frequency	7	1	1–2
Multiband frequency	27	2	1–4
Floatation tank frequency	11	2	1–3
Recommended time from being identified as down to (h):			
Calling the veterinarian	29	7	1–24
Feed/water	30	2	1–6
Medications	29	1	0–12
Relocation	30	1	0–12
Reposition	27	3	0–48
Assisted standing	28	12	1–34

*Veterinarians were asked, with respect to SOPs for down cows, what percent (%) of your producers did the following. **Veterinarians were asked, for the average case, how long is it appropriate to provide general nursing care before recommending euthanasia? ***Veterinarians were asked, how long should assisted lifting methods be used for a down cow before recommendation for euthanasia? The survey was distributed to bovine veterinarians in Ontario and evaluated 5 management areas (cull cows, down cows, male and female dairy calves, antimicrobial use, and disease control and surveillance).

#### Lifting and moving

When asked which options they would consider appropriate for moving down dairy cows, most selected moving a cow via a sled (62.5%) and recommended lifting to assist them in standing using multiband slings (56.3%). Respondents reported the median recommended times for cows to be held up in hip lifters was 5 min for hip lifters (1–35 min), 5 min for belly slings (1–15 min), 15.5 min for multiband systems (3–45 min), and 2 h for flotation tank (0–8 h). Only one respondent provided a recommended time for cows to be held up in ropes per session, which was 35 min. Lastly, respondents recommended a median frequency per day to be lifted by ropes was 2.5 times (range 2–3 times), 2 times for hip lifters (1–3 times), 1 time for belly slings (1–2 times), 2 times for multiband systems (1–4 times), and 2 times for flotation tanks (1–3 times).

#### Feed and water

With respect to providing feed and water, most respondents recommended that down dairy cows have it within reach (58.3, 54.9% respectively). When asked about the frequency for providing feed and water, respondents most commonly recommended that it be available at all times (48.3%).

#### Supportive therapy

When respondents were asked what kind of supportive therapy, they would generally give to down dairy cows, most recommended anti-inflammatory drugs (62.5%). Additional supportive therapy included fluid therapy (33.3%), nutritional supplements (20.8%), reposition therapy (6.3%), antimicrobials (2.1%), and lifting (2.1%). A small number (2.1%) recommended physical exams be conducted as part of ‘supportive care’, likely to ensure ongoing assessment of these cows and any response to the therapies applied.

#### Housing

When asked about what housing options are considered appropriate for down dairy cows, respondents recommended individual pens (40.7%), with 120–250 square feet (53.3%) of space, and bedded with sand (62.5%). Respondents recommended a down dairy cow be positioned “on her chest, supported” (50.0%) and to reposition her (i.e., flip from left to right sided recumbency or vice-versa) every 1–6 h (73.3%).

#### Time from being identified as down to care methods

Respondents’ were asked to provide a recommended amount of time between identifying the cow as down, and various care methods when given the scenario that the cow was found down in sternal recumbency in a scrape alley or free stall barn. In the survey, this was framed as two separate questions, however, these two questions were combined due to the similarity of the responses. The median time suggested by respondents as appropriate for producers to call for veterinary assistance after identifying a down dairy cow was 7 h (1–24 h). Additionally, the respondents recommended an appropriate time of 2 h (1–6 h) from identifying a cow as down until providing feed and water to the cow. Regarding administering medications, respondents advised a median time of 1 h (0-12 h) after identifying the cow as down. Similarly, relocating a down dairy cow was recommended to occur within a median time of 1 h (0–12 h), whereas it was suggested to have a median time of 3 h (0–48 h) and 12 h (1–34 h) for repositioning a down cow and for producers to assist a down dairy cow to stand, respectively.

#### Euthanasia

For the average case, respondents reported a median recommended time span to provide general nursing care to a down cow before recommending euthanasia of 3 days (2–10 d). The median recommended length of time for how long assisted lifting methods should be used for a down dairy cow prior to recommending euthanasia was 3 days (0–7 days).

#### Standard operating procedures

With respect to SOPs for down cows, a median of 72% of the veterinarian’s clients had them view their SOPs (0–100%), while a median of 30% of their clients solicited veterinary input to establish the SOPs (0–100%). Additionally, a median of 50% of respondents reported that their clients had sought their advice when updating their SOPs (0–91%).

### Factors associated with the recommended time to assist a cow to standing

A multivariable linear regression model was developed to identify factors associated with the recommended time to assist a cow to a standing position from finding her down. Table 5 shows the results of the model. Veterinarians who spent >90% of their working hours per year with dairy cattle, recommended that cows should be assisted to a standing position 14.1 h (*p* < 0.01, 95% CI -23.2 to −4.9) earlier when compared to veterinarians who spent ≤85% of their working hours per year with dairy cattle. Respondents at larger clinics (> 7 practitioners who see dairy cattle) recommended a 12.4 h (*p* < 0.01, 95% CI 6.36–18.5) latency until a cow is assisted to stand, than respondents who worked at clinics with less than 3 practitioners who see dairy cattle. Lastly, for respondents who recommended waiting 12–24 h from a cow going down until a veterinarian was called, they recommended cows be assisted to stand 13.8 h (*p* < 0.01, 95% CI 8.1–19.5) longer than veterinarians that recommended waiting ≤7 h from the cow going down until a veterinarian was called.

**Table 5:** Final multivariable linear regression analysis recommendation characteristics associated with the time (hours) from cows being identified as down to assisted standing (*n* = 27).

Variable	Description	Coef.	95% CI	*p*- value
Percentage of working hours spent with dairy cattle in the last 12 months	0–60%	Referent		
	65–85%	−4.1	−12.5 – 4.3	0.32
	90–100%	−14.1	−23.2 – -4.9	<0.01
Number of clinic veterinarians working with dairy cattle	1–3 veterinarians	−1.4	−9.5 – 6.7	0.73
	4–6 veterinarians	Referent		
	7–13 veterinarians	12.4	6.4–18.5	< 0.01
Recommended time from identifying a cow as down to calling the veterinarian (h)	1–7 h	Referent		
	12–24 h	13.8	8.1–19.5	<0.01

### Factors associated with identifying a cow as down to calling a veterinarian

We used univariable linear regression analysis to identify factors associated with the time between identifying a cow as down and calling a veterinarian. Specifically, veterinarians who spent more time working with dairy cattle (≥ 90% of working hours per year with dairy cattle) recommended waiting longer (13.2 h; 95% CI: 6.0–20.4; *p* < 0.01) before calling the veterinarian compared to veterinarians who did less dairy work (≤ 60% of working hours per year with dairy cattle). In addition, for respondents who worked at clinics that serviced ≥80 dairy farms, recommended waiting 9.6 h (*p* = 0.02, 95% CI 1.8–17.4) longer between identifying a cow as down and calling the veterinarian when compared to veterinarians who worked at clinics that serviced ≤40 dairy farms. Lastly, respondents who recommended waiting ≥18 h before assisting a cow to stand, also suggested waiting longer before calling the veterinarian (10.5 h; *p* < 0.01, 95% CI 4.1–16.8) compared to respondents who recommended ≤12 h before assisting a cow to stand. However, when these variables were placed in a multivariable model and screened with a backwards stepwise process, none of the variables were retained, likely due to low power.

## Discussion

This study aimed to shed light on the involvement and perspectives of veterinarians in the management of down dairy cows in Ontario. The evolving role of herd veterinarians, transitioning from individual animal care to providing advice and consultation in farm-level management, underscores the significance of their input in crafting effective management protocols (9).

There was variation in the recommended methods for lifting and moving down dairy cows, perhaps in part due to the case-by-case nature of dealing with down cows. Considerations for lifting and moving may also be based on the available equipment and labor, economic value of the cow, and the producer’s decisions on their management (17). Additionally, devices, such as slings and hip lifters, are considered safe for lifting down dairy cows (18), while moving cows with hip lifters has been shown to greatly increase the risk of additional injuries (6, 18, 19), and is specifically prohibited by proAction and FARM (2, 3) industry programs. Almost all veterinarians in this survey recommended safe methods for lifting and moving down dairy cow, including several veterinarians recommending the use of a flotation tank to lift down cows. Similarly, diverse recommendations were observed for lifting methods with varying time durations and frequencies with respondents recommending a range from 1 to 60 min of time spent in different pieces of equipment and 1 to 4 times per day for lifting. Huxley (17) recommends that animals can be lifted and supported four or more times daily. On the other hand, Stull et al. (18) suggest that hip lifters may be tolerated for 10–15 min twice daily, or slings can be used for several hours several times a day. These wide ranges for lifting time and frequency emphasize the need for evidence-based guidelines and consensus within the veterinary community.

In our study, 62% of veterinarians recommended providing NSAIDs to down cows, a practice supported by research to improve welfare and recovery (20, 21). Administering NSAIDs may be beneficial for most down dairy cows by alleviating pain and reducing inflammation (17, 22–24). Beyond NSAIDs, additional supportive therapies, such as fluid therapy, nutritional supplements, reposition therapy, bloodwork, antibiotics, lift therapy, and physical exams, were recommended. Understanding the cause of recumbency is vital for determining the appropriate additional treatments for down dairy cows (17, 25). However, diagnosing the exact cause of recumbency can be challenging as clinical symptoms alone are often insufficient (25). This highlights the importance of tailoring therapy recommendations based on understanding of the individual cow’s condition.

Timely interventions and diligent nursing care are crucial factors in improving the prognosis and overall well-being of down dairy cows, as emphasized by research (5, 17). Veterinarians commonly recommended providing essential care, including feed and water, medications, relocation, and repositioning, within 5 h of identifying a cow as down, based on data presented in this study. Studies by Poulton et al. (5) and Stojkov et al. (6) demonstrate the significant impact of early high-quality nursing care on the recovery of down dairy cows. Poulton et al. (5) categorized nursing care into a four-tiered grading system and found a strong association between the level of care provided and cows’ recovery. The four tiers, ranged from poor care with infrequent repositioning and inadequate bedding (Tier 1) to exemplary care with frequent repositioning, meticulous hygiene, and proactive monitoring to prevent complications (Tier 4) (5). Our multivariable regression analysis expands on this by emphasizing the importance of timely and high-quality nursing care while also identifying key factors influencing veterinarians’ recommendations for assistance times. Our model identified several factors influencing the recommended time for assisting down dairy cows. Veterinarians who spent more than 90% of their working hours with dairy cattle recommended shorter response times for assisting a cow to stand. This may reflect greater exposure to down cows and more experience, leading to a preference for prompt intervention. Conversely, veterinarians working in larger clinics recommended longer response times for assistance, although the underlying reason for this association remains unclear. Additionally, we observed that longer recommended wait times before calling a veterinarian were associated with longer assistance times. Similarly, Stojkov et al. (6) observed that cows receiving poor nursing care were less likely to recover. Despite these recommendations for higher quality care, producers often fall short in implementing them (4). Various factors, including economic constraints, labor availability, and the specific etiology of recumbency, likely contribute to this gap between veterinary recommendations and actual nursing care practices (6, 26, 27). This gap underscores the need to explore the broader context of veterinary workload and clinic dynamics, as discussed in the next section.

A study by Ballantyne and Buller (28), discussed workload issues faced by veterinarians in clinical behavioral medicine, including the impact of clinic size on their experiences and workload challenges. The study elaborates on these challenges including the need for more support staff, time demands, paperwork, income concerns, and positive career attributes (28). Additionally, they suggested that clinic size and the composition of the veterinary team may influence workload and practice dynamics in veterinary behavior clinics (28). Our findings could be the result of similar factors, however, further research to better understand these factors in the dairy industry is needed.

Multiple housing and bedding options were considered appropriate for down dairy cows by respondents in this study; however, most recommended that down dairy cows should be housed in individual stalls with sand bedding. Moreover, it was commonly recommended that clean water and feed be available within an easily accessible distance. This is in line with recommendations by Poulton et al. (5) and proAction requirements of providing feed and having water available every hour for down dairy cows (2).

There are concerns in the literature regarding the decision-making process for euthanasia (29). Decisions surrounding euthanasia can be a complicated and multi-factorial process, and more information and training are commonly needed on farms (17, 29–31). Training programs and more specific euthanasia standards can improve welfare and ensure consistent euthanasia decision-making, particularly given the lack of consensus among dairy veterinarians regarding conditions and timelines for euthanasia (29–31). Cows may suffer unnecessarily if not euthanized soon enough, as well as the possibility of cows that have a chance at recovery being euthanized too early (31). In this study, respondents generally recommended providing 3 days of nursing care before making the decision to euthanize the cow. Additionally, the mean recommended time for the time spent using lifting methods before euthanasia was also 3 days, with a range of 0–7 days. McFarlane et al. (1) found that some producers waited an average of 4.5 days before deciding to euthanize a down cow, whereas another study by Wagner et al. (29) demonstrated that some producers do not ever choose to euthanize animals, highlighting the potential discrepancy between producers and veterinarians. These studies underscore the need for greater consensus and the complexity of making the decision to euthanize a down cow.

This study has limitations that may impact the generalizability of the results. The survey’s length may have contributed to participant drop-off, with some respondents not answering questions relevant to our topic. Additionally, the section on down cow management was positioned midway through the survey, which could have affected engagement with response rates for questions related to down cow management ranging from 58 to 100%.

Additionally, due to the data being from 2021 and a 26.8% response rate from OABP members, these data may only partially reflect the recommendations of Ontario dairy veterinarians in 2024. Additionally, not having paper copies of the survey may have affected the demographics of participants. Despite the acknowledged limitations of this study, the results still provide valuable insights into current practices in down cow management on Ontario dairy farms. Moving forward, collaborative efforts between veterinarians, researchers, and dairy producers are essential to develop evidence-based protocols that ensure down cows’ optimal care and well-being in diverse farming environments.

This study examined the down dairy cow management practices employed by Ontario veterinarians, shedding light on crucial aspects that could influence the adoption of care approaches. A more uniform and evidence-based approach from veterinarians could contribute to improved down dairy cow care and align with the broader goal of advancing effective management protocols.

## Data Availability

The data presented in this study are deposited in the Borealis repository, accession number https://doi.org/10.5683/SP3/PFPUDO.

## Footnotes

^1^ https://doi.org/10.5683/SP3/PFPUDO
